# Analysis and Evaluation of Load-Carrying Capacity of CFRP-Reinforced Steel Structures

**DOI:** 10.3390/polym16182678

**Published:** 2024-09-23

**Authors:** Jian Zhao, Yongxing Huang, Kun Gong, Zhiguo Wen, Sinan Liu, Yanyan Hou, Xuewu Hong, Xuecheng Tong, Kai Shi, Ziyi Qu

**Affiliations:** 1 School of Control and Mechanical Engineering, Tianjin Chengjian University, Tianjin 300384, China; zhaojian_tju@163.com (J.Z.); hyx990919@outlook.com (Y.H.); gongkun1998@outlook.com (K.G.); wenzhiguo_0709@foxmail.com (Z.W.); liusinan@tcu.edu.cn (S.L.); hxw789164@163.com (X.H.); quziyi98@outlook.com (Z.Q.); 2 Tianjin Puret Purification Technology Co., Ltd., Tianjin 301814, China; qiaolizh@aliyun.com; 3 School of Mechanical Engineering, Tianjin University, Tianjin 300354, China; sk19940502@tju.edu.cn

**Keywords:** carbon fiber-reinforced polymer, steel structure, structural reinforcement, load-carrying evaluation, random forest

## Abstract

Carbon Fiber Reinforced Polymer (CFRP) can be used to reinforce steel structures depending on its high strength and lightweight resistance. To analyze and evaluate the load-carrying capacity of CFRP-reinforced steel structures. This study uses the Finite Element Analysis (FEA) and the experimental tests combined to investigate the influence that the reinforcement patterns and the relevant parameters have on the load-carrying capacity. We made specimens with different reinforcement patterns. Take the steel beam specimen with full reinforcement as an example. Compared with the load-carrying capacity of the steel beam reinforced by two-layer CFRP cloth, that respectively increases by 5.16% and 11.1% when the number of the CFRP cloth increases to four and six, respectively. Based on a specimen set consisting of CFRP-reinforced steel structures under different reinforcement patterns, the random forest algorithm is used to develop an evaluation model for the load carrying. The performance test results show that the MAE (Mean Absolute Error) of the evaluation model can reach 0.12 and the RMSE (Root Mean Square Error) is 0.25, presenting a good prediction accuracy, which lays a solid foundation for the research on the CFRP-based reinforcement technology and process.

## 1. Introduction

Steel structures are widely used in bridges, high-rise buildings, and heavy-duty plants due to their high strength, good seismic resistance, and short construction period [1,2,3]. However, steel structures in service are inevitably affected by various vibrations, impact or alternate loads, and corrosion, resulting in failing to reach their designed carrying capacity limit and structural safety. Traditional reinforcement methods for steel structures, such as welding and riveting, often cause local stress concentration, which increases the risk of secondary damage. As a new reinforcement method for steel structures, CFRP-based reinforcement is proven to be economical and efficient since it does not need to change the original structure and add new weld seams, avoiding the potential risk of secondary damage associated with the residual stress and new fatigue-sensitive sources.

At present, the CFRP-based reinforcement method has become a hotspot in the relevant field, and lots of research has paid attention to appraising the performance and effect of the CFRP cloth on the load-carrying capacity of the steel structure. Elkhabeery et al. [4] used CFRP to strengthen and repair bent steel girders, and the results show that the CFRP sheets are very effective in strengthening compact uniaxially symmetric sections, whereas non-compact section girders had a very small effect on the strengthening. Bastani et al. [5] utilized CFRP cloth to repair damaged I-beams, and the results show that the load-carrying abilities of the damaged beams can be restored to a similar level of the beam without damage. Kypriadis et al. [6] studied the influences of the patch thickness and length on the load carrying of the CFRP-inforced H- and square-shaped steel girders. Chen et al. [7] studied the mechanical performance of marine concrete with carbon fiber-reinforced polymer (CFRP)-aluminum alloy tube columns (CFRP-CFAT), which presents increased load-carrying capacity and energy absorption ability. Han et al. [8] analyzed the damage evolution and patterns of the CFRP laminates with different bond line thickness adhesives. Although lots of research proves that the CFRP can effectively reinforce the steel structure and improve the load-carrying capacity of the steel structure, the reinforcement effect is closely related to many factors, and the relationship is nonlinear, which results in the difficulty in developing the mapping models; therefore, there is a necessity to investigate the influential factors and relevant rules and give a method to accurately evaluate the reinforcement effect.

To develop the mapping model describing the nonlinear relationship between the factors of the CFRP cloth and the load-carrying capacity of the reinforced steel structure, machining learning methods are extensively used. Mojtabaei et al. [9] combined the Finite Strip Method (FSM), Equivalent Nodal Force Method (ENFM), and multilayer artificial neural network (ANN) to evaluate the elastic critical buckling loads. Kourosh et al. [10] used a Fuzzy Inference System (FIS) to establish a more accurate and reliable method for predicting the shear strength of CFRP-reinforced concrete. Gia et al. [11] utilized three kinds of algorithms to predict the punching shear strength of the CFRP floor. Viet-Linh et al. [12] provided an accurate prediction of punching shear strength for bi-directional reinforced concrete slabs using the designed artificial neural network model. Khan et al. [13] used artificial neural network (ANN) and random forest (RF) regression to estimate the flexural capacity of beams and achieved good prediction accuracy. Hu et al. [14] applied Machine Learning to optimize the properties of CFRP-reinforced metal composites and developed the gradient boosting models to evaluate the Extreme Gradient Boosting (XGBoost) and gradient boosting models, and the results demonstrate that the developed method can predict the tensile strength well.

Although using machining learning to evaluate the performance of CFRP reinforcement can have a certain effect, this kind of method commonly needs large samples, and it is costly and time-consuming. Moreover, the method has to face many problems, such as the curse of dimensionality, overfitting, and so on. To develop a more concise evaluation method under small-size dataset conditions, this research carefully evaluates the load-carrying capacity of CFRP-reinforced beams using a synergistic framework of experiments and simulations. Through comparative analyses, the determining factors affecting the load-carrying capacity of the structure are identified, and the results of reinforcement under different factor arrangements are scrutinized. Subsequently, a reliable numerical simulation model for the load-carrying capacity of CFRP-reinforced steel beams is developed. The load-carrying capacity prediction using the random forest algorithm under small size dataset conditions is implemented.

## 2. Materials and Methodology

### 2.1. Materials and Fabrication of the Specimens

**Figure 1:**
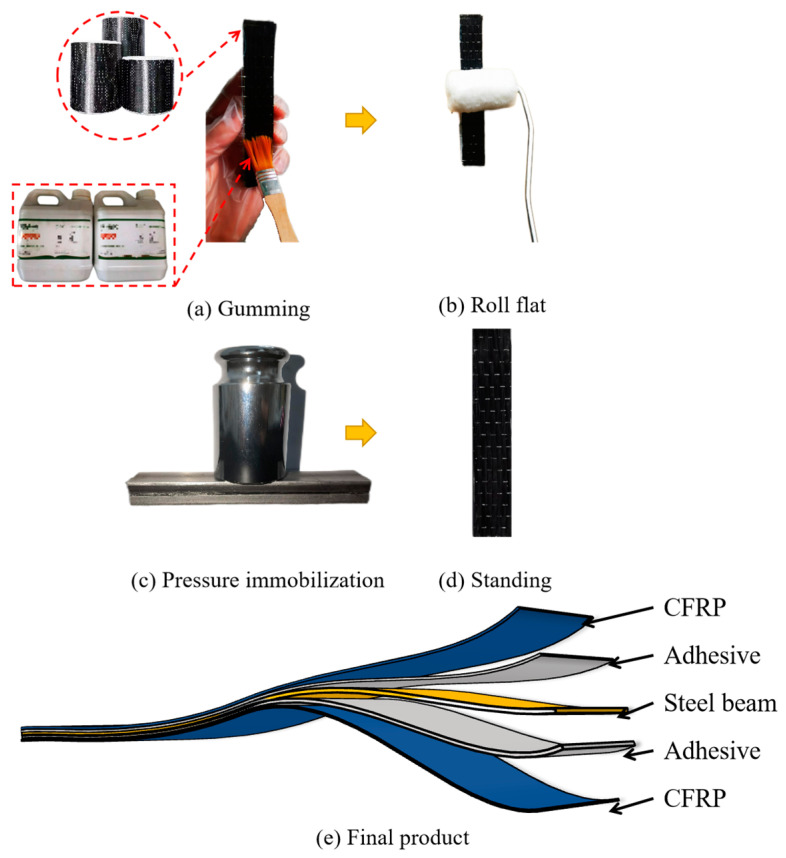
Fabrication of the CFRP-reinforced steel beam specimens.

The fabrication of specimens required to bond the CFRP cloth on the steel beam layer by layer. Here, a single-layer CFRP-reinforced steel slab is taken as an example to introduce the fabrication process. The specimens are fabricated with Q235, which is reinforced by CFRP cloth. The important parameters for specimen fabrication are shown in Table 1. The main steps of the fabrication of specimens are gumming, rolling flat, pressure immobilization, and standing, as shown in Figure 1a–d. To ensure the thickness consistency of the adhesive layer between the steel beam and the CFPR cloth, steel balls with different diameters were used through laying evenly in the adhesive layer. Then, the 500 g weights are uniformly distributed on the CFRP layers to maintain the stability of the adhesive layers for at least 5 h. Finally, the specimens are placed in the incubator and cured at 30 °C for 7 days. The final structure of the fabricated single-layer CFRP-reinforced steel beam specimen is shown in Figure 1e.

**Figure 2:**
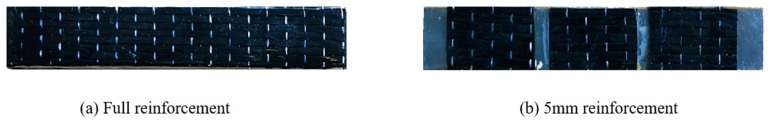
Photos of the fabricated specimens: (**a**) four-layer full-bond reinforcement and (**b**) 5 mm reinforcement.

Two kinds of fabricated CFRP-reinforced steel specimens adopting four-layer CFRP cloth are respectively shown in Figure 2a,b, including a specimen of full-bond reinforcement and a specimen of partial-bond reinforcement with a 5.0 mm interval between two CFRP blocks. For the convenience of expression, full-bond reinforcement is flagged as full reinforcement, and partial-bond reinforcement with a 5 mm interval is flagged as 5 mm reinforcement.

### 2.2. Load-Carrying Test Trial

**Figure 3:**
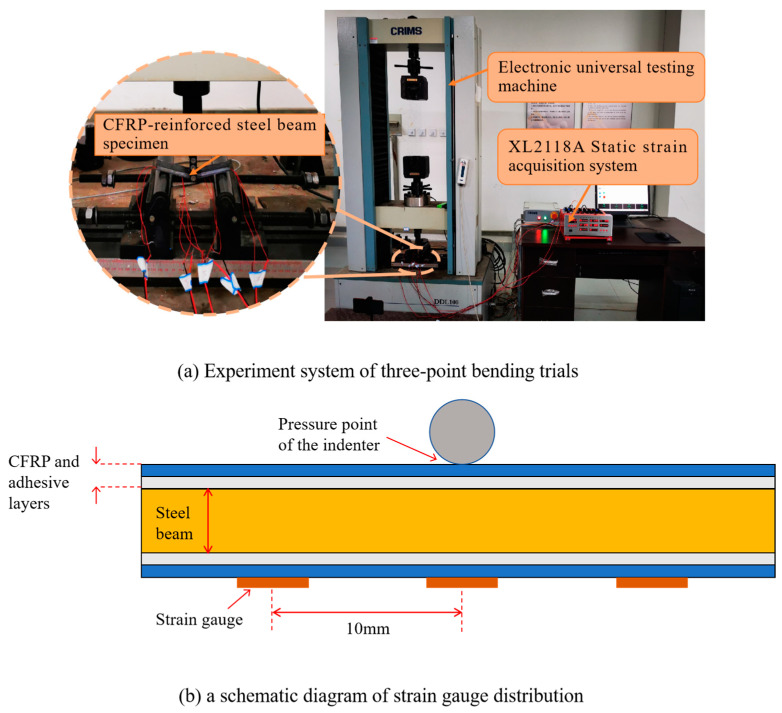
(**a**) Experiment system of three-point bending trials and (**b**) a schematic diagram of strain gauge distribution.

To test the load-carrying capacity of the CFRP-reinforced steel beam specimens, three-point bending trials are carried out [15]. The experimental system is shown in Figure 3a. An electronic universal testing machine (DDL100., Changchun Institute of Machinery Science, Changchun, China) is used to produce the pressing force on the specimens, and the load-carrying of the specimens is measured by three BX120-3AA strain gauges (the size of the strain gauges is 5 mm × 3 mm, the resistance is 120 Ω, the sensitivity coefficient is 2.08 ± 1%, and the measurement range is from 0 to 19,999 με) and XL2118A static strain gauge. The layout of the strain gauges is shown in Figure 3b, among which one is centered just under the indenter of the DDL100 machine, and the other two gauges are placed symmetrically on either side of a 10 mm interval. Specimen production and test procedures meet ASTM D7264/D7264M-21 standards [16].

**Figure 4:**
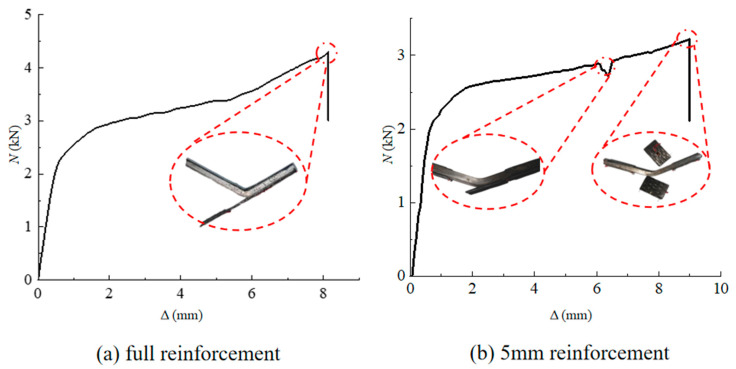
Deformation of the CFPR-reinforced beam versus the applied load, (**a**) four-layer full-bond reinforcement and (**b**) 5 mm reinforcement.

During the three-point bending trials, a pressing force is applied at a velocity of 2 kN/s. The test is stopped when the specimen fails in complete bending. The relationship between the deformation of the four-layer full reinforcement specimen and the force is shown in Figure 4a. It is evident that the beam specimen begins to bend when the load reaches 2 kN, and the lower left side of the CFRP cloth slips along the beam surface. As the pressing force increases, the beam further bends, and the lower side of the CFRP cloth peels away from the beam at 4.297 kN.

The load-carrying of the steel beam reinforced by four-layer 5 mm reinforcement is shown in Figure 4b. When the pressing force is 2.8 kN, the CFRP cloth of the bent steel beam peeled away from the steel beam, and the fiber partially broke with a consequent decrease in the pressure transducer. The CFRP slipped, and the peeling phenomenon is gradually aggravated as the load continues to increase. With the load increased to 3.924 kN, part of the carbon fiber is completely detached, and the specimen failed.

The load-carrying capacity of a 6 mm steel beam without reinforcement is 2.8 kN [17], and that of the four-layer full reinforcement and 5 mm reinforcement was increased by 43.23% and 30.80%, respectively. The results show that the CFRP cloth can effectively improve the load-carrying capacity of the steel beam specimen, and the reinforcement effect will be affected by different factors.

## 3. Results and Discussion

### 3.1. Specimen Design

Generally, the load-carrying capacity of the CFRP-reinforced steel beam can be affected by many factors, among which the number of CFRP layers, adhesive layer thickness, and the CFRP block interval are proved significant [18,19]. The CFRP block interval is directly referred to as the reinforced interval. In order to master the law of increasing load-carrying, it is necessary to study different influencing factors of reinforcement.

To investigate the influential rules of these factors, a series of specimens under different reinforcement patterns are fabricated and divided into two groups.

**Figure 5:**
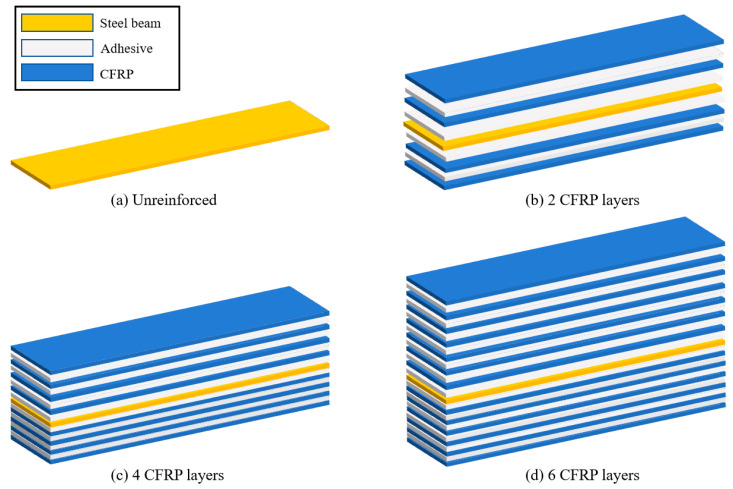
Steel beam specimens reinforced with different CFRP layers.

Specimens in the first group to analyze the effect of the number of CFRP layers. The group has the same adhesive layer thickness of 0.3 mm and a different CFRP cloth layer number, including two-, four-, and six-layers, as schematically shown in Figure 5.

**Figure 6:**
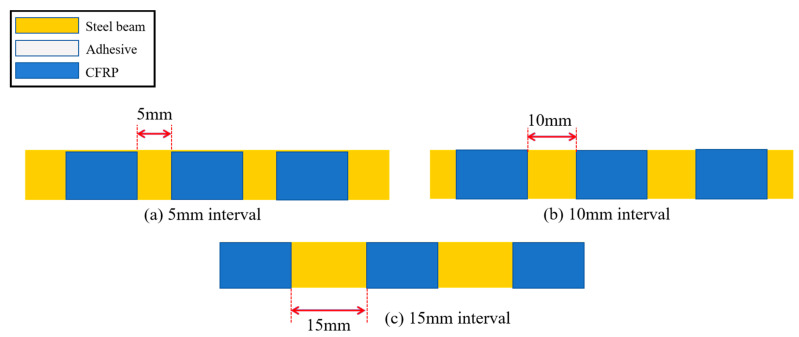

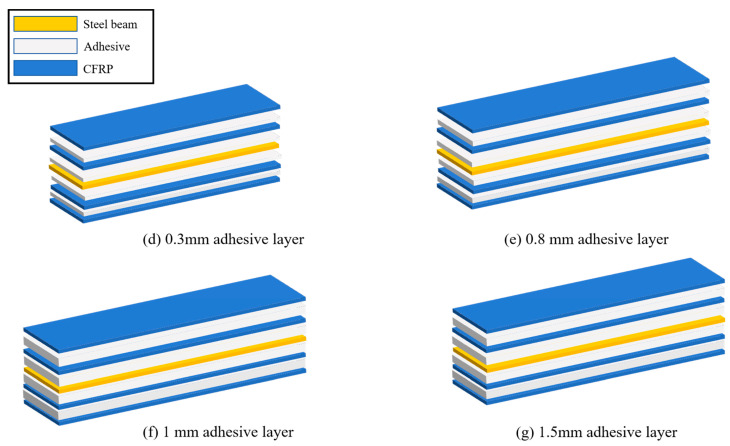
Steel beam specimens with different intervals and different adhesive layers.

Beam specimens of the second group are all reinforced by two layers of CFRP cloth, with the thickness of the beams being 6 mm. In order to study the effect of the reinforcement interval on the load-carrying of the steel beams, the specimens are designed with reinforcement intervals of 5 mm, 10 mm, and 15 mm, as shown in Figure 6a–c. The specimens with full reinforcement of CFRP cloth are designed with adhesive layer thicknesses of 0.3 mm, 0.8 mm, 1.0 mm, and 1.5 mm to investigate the effect of adhesive layer thickness on the load-carrying capacity of steel beams, as shown in Figure 6d–g.

### 3.2. Analysis of Experimental Data from Three-Point Bending Tests

**Figure 7:**
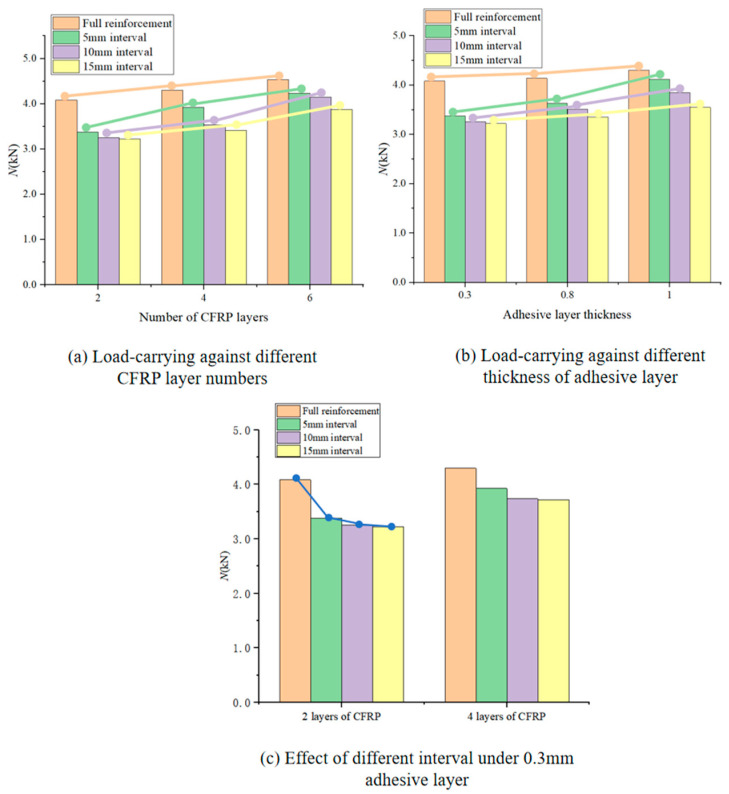
Comparison of load-carrying capacity of steel structures under different reinforcement methods.

Each group of specimens was subjected to three load-carrying capacity experiments. The average of the three experimental results was taken to be presented in the paper under the premise of controlling the consistency of experimental conditions. The small deviation of the results for each group of specimens proves that the experimental results have high repeatability. The final comparison of the load-carrying capacity of the steel structure obtained with different reinforcement methods is shown in Figure 7.

The effect of the CFRP cloth number on the load-carrying is shown in Figure 7a, and it can be seen, for all reinforcement patterns, that the load-carrying capacity of the steel beam increases with the increase in the CFRP layer number. Take the steel beam specimen with full reinforcement as an example. Compared with the load-carrying capacity of the steel beam reinforced by two-layer CFRP cloth, that respectively increases by 5.16% and 11.1% when the number of the CFRP cloth increases to four and six, respectively. The influence of the thickness of the adhesive on the load-carrying capacity of the steel beam specimens is shown in Figure 7b. The load-carrying increases significantly with an increased thickness. For the beam specimen adopting full reinforcement of two-layer CFRP, it has a load-carrying capacity of 4.082 kN when the adhesive layer thickness is 0.3 mm, but it respectively increases to 4.144 kN and 4.3 kN when the adhesive layer is 0.8 and 1 mm in thickness.

For the steel beam specimens reinforced by two- and four-layer CFRP cloth, their load-carrying changes versus the reinforcement pattern are shown in Figure 7c. It can be seen that the load-carrying capacity of the beam specimens presents a notable decrease tendency as the interval between two CRFP blocks changes from 0 (full reinforcement) to 15 mm. There is an interesting phenomenon: the load-carrying capacity of the steel beam specimen with full reinforcement is significantly greater than that of the specimen from partial reinforcement; however, for the specimens from partial reinforcement, their load-carrying capacity only decreases slightly with the increase in the interval. This is to say, in the actual application, that the interval should be reasonably selected according to the construction requirements to avoid unnecessary waste.

### 3.3. Comparison of the Influential Factor of the Load-Carrying

To analyze and compare the influence degree of the three factors mentioned above on the load-carrying capacity of the beam specimen. Combined consideration of test objectives and test efficiency, a group of orthogonal tests under three-factor and three-level is carried out. Before the test, the steel beam specimens are fabricated according to the orthogonal test table (Table 2) [20,21,22].

The results of the range analysis and variance analysis are listed in Table 3 and Table 4. Compared with the CFRP layer number and adhesive layer thickness, the reinforced interval has the biggest F-value, indicating it has the most significant influence on the load-carrying capacity of the CFRP-reinforced steel beam. CFRP layer number has a greater influence on the load-carrying capacity compared with the adhesive layer thickness.

## 4. Load-Carrying Capacity Evaluation

### 4.1. Finite Element Analysis of the CFRP-Reinforced Steel Beam

To solve the problem of time-consuming consumables for the test. The FEA model is used to analyze the load-carrying capacity of CFRP-reinforced steel beams to save time and reduce cost. The FEA results can be verified for correctness through the experimental results [23,24,25].

#### 4.1.1. Selection of Unit Type

1. Steel Beam

In the simulation of steel beams, the 3D solid element C3D8R from the ABAQUS 6.14 library is utilized.

When the mesh of C3D8R is twisted and deformed, the accuracy of the analysis remains largely unaffected, making it highly suitable for analyzing components that undergo large deformations.

2. CFRP Plate

When simulating Carbon Fiber Reinforced Polymer (CFRP), it is essential to mitigate the impact of the thickness disparity between the steel beam and the CFRP model. To achieve this, the S4R elastic shell element, which possesses both bending and membrane characteristics, is employed to represent the CFRP plate. This element is a four-node elastic shell unit, with each node exhibiting six degrees of freedom: three translational displacements in the respective directions and three rotational displacements about each axis [26].

3. Adhesive Layer

The adhesive layer is represented by an eight-node three-dimensional cohesive element, COH3D8. This cohesive element is a viscous unit designed to simulate the viscous connection between two components.

#### 4.1.2. Grid Division

CFRP employs free meshing, which is the most flexible mesh division technology available and is applicable to a wide range of models. The three-dimensional cohesive unit COH3D8 selected for the adhesive layer does not require mesh refinement; therefore, the adhesive layer utilizes a swept mesh. Given the significant deformation of the steel plate, a structured network is employed. Structured network technology (STRUCTURE) standardizes the grid pattern configuration in uncomplicated and simple geometric areas. This method displays different colors to distinguish between various mesh division techniques across the mesh area.

#### 4.1.3. Boundary Conditions

According to the experimental design of CFRP-reinforced steel plates, two reference points, RP-1 and RP-2, are established at the left and right ends of the plate. These reference points are coupled with the end plates using point-face coupling in ABAQUS. This method not only facilitates the application of the corresponding constraints but also prevents damage to the cross-section in the local areas under conditions of concentrated load. When a load is applied, if it is directly imposed on the steel beam, the stress at the loading point may become excessively high, leading to difficulties in achieving convergence in calculations. To address this, a loading column is first established with a central reference point, which is then coupled and configured as a rigid body. All degrees of freedom, except for those in the direction of the loading end, are constrained at the loading end. The load is applied at the reference point to effectively transfer the load to the beam. The load displacement is applied at the loading end in a manner that ensures the convergence of the calculations.

#### 4.1.4. Comparison of Numerical Analysis and Experimental Results

**Figure 8:**
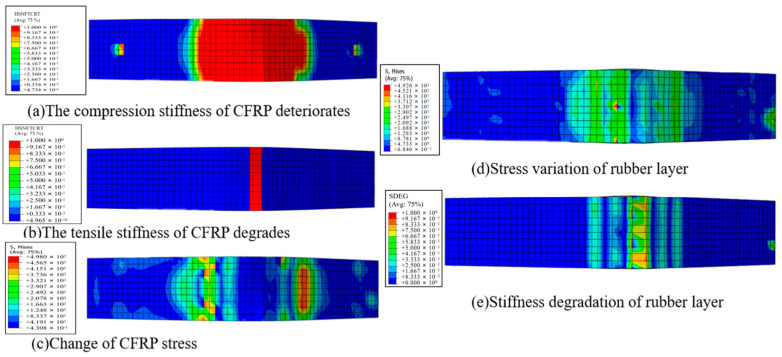
Damage evolution process of full reinforcement steel beam.

The deformation and damage of the CFRP-reinforced steel beam against the increased pressing force are shown in Figure 8. As shown in Figure 8a, with the increases in the load, fiber of the CFRP layer compression damage occurs; the stiffness of the fibers in compression damage is reduced by 100%, resulting in complete failure of the fibers. The CFRP may undergo either fracture damage or peeling damage and loss of reinforcement effect [27].

As shown in Figure 8b, as the load continues to increase until the fiber tensile damage occurs, the stiffness of the fiber compression damage decreases. The fiber is completely failed by tension; CFRP may cause tearing damage or peeling damage, resulting in the loss of the reinforcement. At this time, the adhesive layer is also completely damaged, as shown in Figure 8e.

As shown in Figure 8c,d, it can be seen that the stress concentration easily occurs in the compressed area of the CFRP layer, which makes the uneven distribution of the stress along the length direction. That can result in the peeling damage of the CFRP layer and adhesive layer.

**Figure 9:**
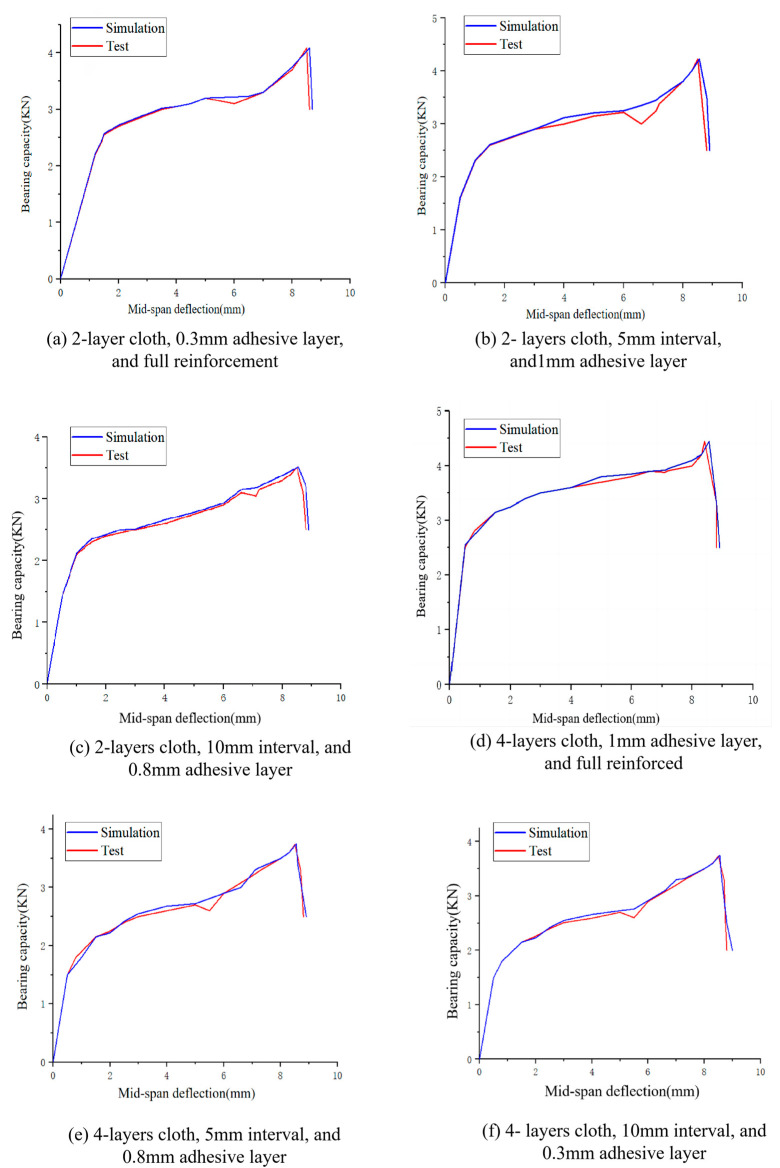
Comparison of FEA and experimental results.

**Figure 10:**
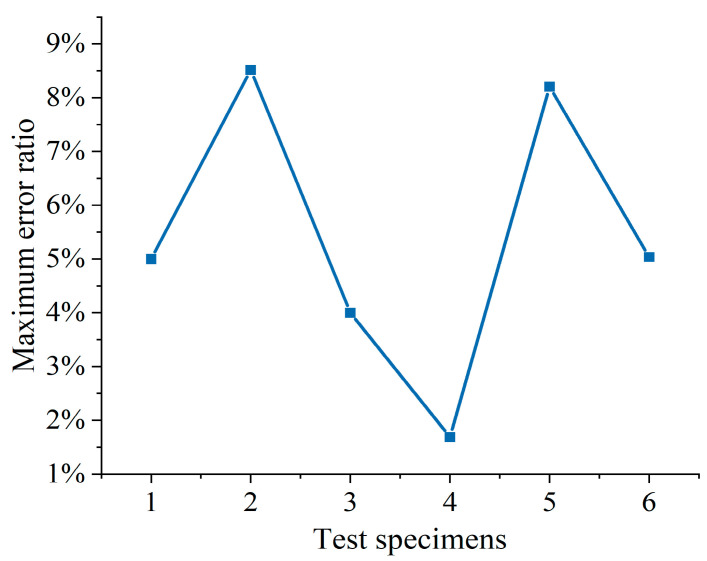
Maximum error ratio.

The results of the FEA and experimental test are compared in Figure 9 and Figure 10. The maximum error between the test and the simulation is 0.23 kN, and the average maximum error of the six comparisons is within 0.15 kN. The maximum error is within 10% of the test reference value. It can be concluded that the simulated maximum value and the measured maximum value are basically the same, and the established FEA model has good reliability.

### 4.2. Establishment of Dataset Based on FEA Analysis

To develop a load-carrying capacity prediction model, a training dataset is built based on FEA analysis, as shown in Table 5, where a total of 33 specimens are listed.

### 4.3. Establishment of Prediction Model of Load-Carrying Capacity of CFRP Reinforced Steel Beams

The random forest method is used to predict the load-carrying capacity of CFRP-reinforced steel beams based on the small datasets established by FEA [28,29,30]. Random forest maintains high accuracy even with small datasets.

Compared with most machine learning methods, the random forest has strong prediction capability and is fast both in training and predicting. Generally, the random forest adopts an ensemble training algorithm. Through a random selection of the training specimens to construct multiple decision trees, the risk of overfitting can be suppressed. A similar way is also done in bagging, making the construction of the prediction tree robust against noise. Moreover, during the splitting of the nodes, the features are also selected randomly, enabling a fast training process, even for the feature vector with a large dimensionality.

Tuning Optimization is based on GridSearchCV for random forest models. Generally, the algorithm prediction accuracy is characterized by being reflected by the Root Mean Square Error (RSME) value. The prediction accuracy of the algorithm is increased as the RSME value decreases. The model performance is closely related to the decision tree numbers in the forest; here the number is flagged as *n-estimators*(*n*).

**Figure 11:**
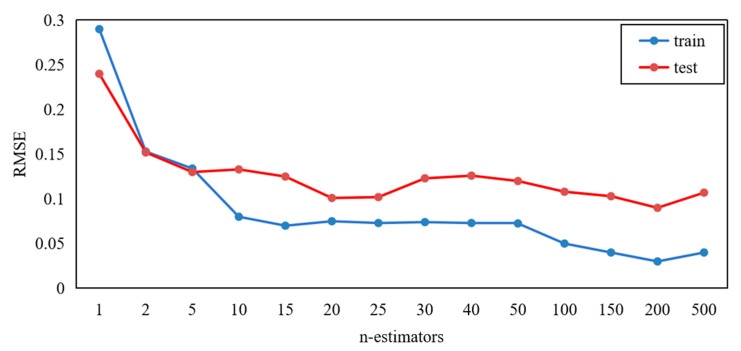
Parameter optimization.

As can be seen in Figure 11, the RMSE of the test dataset is lowest when the *n* = 20. Thus, the random forest algorithm *n* = 20 is selected to be the optimal parameter. The prediction accuracy does not exceed this peak value as the number of decision trees increases. Therefore, the number of the decision trees is determined as 100. The selection of 20 decision trees in the random forest is ideal in the experimental data collected, considering the number of decision trees to the computational speed of the prediction model.

In order to compare the excellence of other common prediction methods and random forest models and verify the accuracy of random forest in small datasets, this paper uses open data sets to build test sets to test the KNN model, decision tree model, and random forest model. In the test set, 40 groups are randomly selected from the 638 groups of data provided [31]. The performance of each model in the test set is shown in Table 6.

According to the comprehensive evaluation of the evaluation indicators, it can be concluded that the random forest model can maintain sufficient accuracy and is superior to other comparison models under small dataset.

### 4.4. Validation of Load-Carrying Capacity Prediction Model for CFRP Reinforced Steel Beams

The cross-validation method is used to verify the correctness of the load-carrying capacity model for CFRP-reinforced steel beams [32]. Cross-validation is a common method to verify the reliability of the model, which can avoid the bias caused by randomly dividing the training set by dividing the data into *n* parts. One of them is used as the test set in turn, and the other *n* − 1 parts are used as the training set. The accuracy of the model is calculated several times to evaluate the average accuracy of the model.

**Figure 12:**
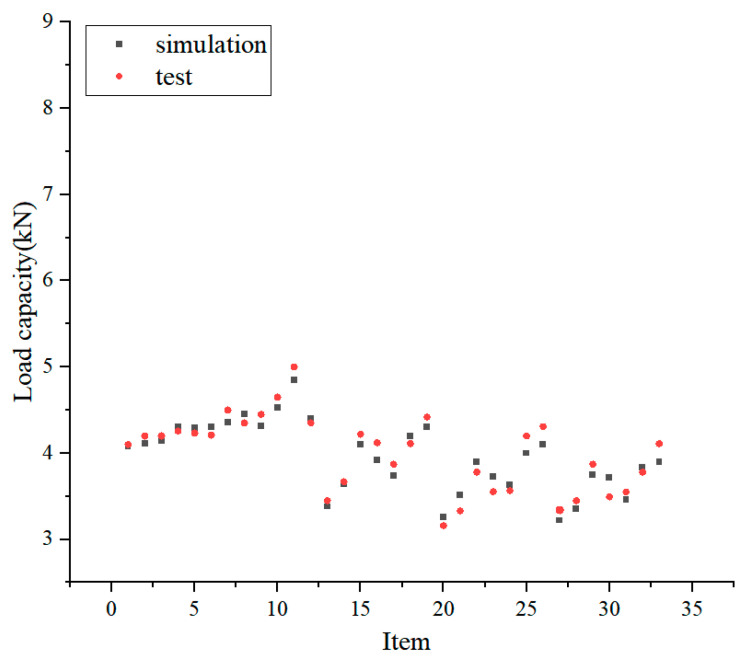
Load-carrying capacity evaluation results.

The comparison between the measured and predicted values of the load-carrying capacity of CFRP-reinforced steel beams is shown in Figure 12. It can be seen that the predicted and measured results are in good agreement.

The prediction model results are compared with the experimental results, and the prediction accuracy of the prediction model is evaluated using indexes of the mean absolute error, root mean square error, and determination. According to the calculations, the Mean Absolute Error (MAE) is 0.12, the RMSE is 0.25, and the R^2^ is 0.96, demonstrating that the prediction model can work well. Compared with other methods for predicting (e.g., the XGBoost method in an existing study), the MAE and RMSE values of the XB model were 0.058 and 0.099, respectively [33]. The random forest algorithm with small samples is sufficient to meet the engineering accuracy requirements in a comprehensive comparison.

## 5. Conclusions

In this paper, an experimental method combined with a machine learning method is proposed to predict the load-carrying capacity of CFRP-reinforced steel beams. The prediction accuracy of the prediction model is evaluated through the experiment results. The results show that the method proposed in this paper can effectively predict the carrying capacity of CFRP-reinforced steel beams with a small dataset, which helps to reduce the time and costs of load experiments. The main conclusions are obtained as follows:

1. It is analyzed that the reinforcement interval has the greatest influence on the load-carrying capacity of CFRP-reinforced steel beams, followed by the number of CFRP layers, and the thickness of the adhesive layer had the lowest influence. Their F-values were calculated to be 5.010, 2.33, and 0.863, respectively.
2. The prediction model for the load-carrying capacity of CFRP-reinforced steel beams is established based on the random forest algorithm with small datasets. The MAE and RMSE values of the prediction model were verified to be 0.12 and 0.25, respectively, which prove the effectiveness of the prediction algorithm.
3. The use of a validated FEA model to build the dataset for the prediction algorithm of load-carrying capacity effectively saves spending on experiments.

## Figures and Tables

**Table 1:** Important parameters of the specimen.

Components of Specimen	Parameters
Steel beam	Material: Q235 Long: 120 mm wide: 20 mm thickness: 6 mm
CFRP layer	Material: 37 12 k carbon fiber filaments thickness: 1 mm
Adhesive layer	Bending strength: 90.8 MPa Compressive strength: 89.5 MPa

**Table 2:** Specimens used in the orthogonal test.

CFRP Layers	Reinforcement Interval (mm)	Thickness of Adhesive Layer (mm)	Load-Carrying Capacity (kN)
2	0	0.3	4.082
2	5	1	4.120
2	10	0.8	3.512
4	0	1	4.446
4	5	0.8	3.736
4	10	0.3	3.733
6	0	0.8	4.852
6	5	0.3	4.120
6	10	1	4.100

**Table 3:** Range analysis results.

Item.	CFRP Layers	Reinforcement Interval	Thickness of the Adhesive Layer
K1	11.714	13.380	11.935
K2	11.915	11.976	12.100
K3	13.072	11.345	12.666
k1	3.905	4.460	3.979
k2	3.972	3.992	4.033
k3	4.357	3.782	4.222
Range	0.453	0.678	0.244

**Table 4:** Variance analysis results.

Source of Variance	Sum of Squares of Deviations	Degree of Freedom	Mean Square	F-Value	*p*-Value
CFRP layers	0.332	2	0.166	2.34	0.299
Reinforcement interval	0.711	2	0.356	5.011	0.166
Thickness of adhesive layer	0.124	2	0.061	0.863	0.537

**Table 5:** Simulated data on load-carrying capacity of CFRP-reinforced steel beams.

Thickness of Steel Beams (mm)	Interval (mm)	Number of CFRP Layers	Adhesive Layer (mm)	Force (kN)	Increase (%)
6	0	2	0.30	4.08	36.07
6	0	2	0.50	4.11	37.00
6	0	2	0.80	4.14	38.00
6	0	2	1.00	4.30	43.33
6	0	2	1.50	4.29	43.00
6	0	4	0.30	4.30	43.23
6	0	4	0.80	4.36	45.27
6	0	4	1.00	4.45	48.20
6	0	4	1.50	4.31	43.60
6	0	6	0.30	4.53	51.10
6	0	6	0.80	4.85	61.73
6	0	6	1.00	4.40	46.67
6	5	2	0.30	3.38	12.50
6	5	2	0.80	3.64	21.20
6	5	2	1.00	4.10	36.67
6	5	4	0.30	3.92	30.80
6	5	4	0.80	3.74	24.53
6	5	4	1.00	4.20	40.00
6	5	6	1.00	4.30	43.33
6	10	2	0.30	3.26	8.53
6	10	2	0.80	3.51	17.07
6	10	2	1.00	3.90	30.00
6	10	4	0.30	3.73	24.43
6	10	4	0.80	3.63	21.00
6	10	4	1.00	4.00	33.33
6	10	6	1.00	4.10	36.67
6	15	2	0.30	3.22	7.40
6	15	2	0.80	3.35	11.77
6	15	2	1.00	3.75	25.00
6	15	4	0.30	3.72	23.90
6	15	4	0.80	3.46	15.33
6	15	4	1.00	3.83	27.67
6	15	6	1.00	3.90	30.00

**Table 6:** The performance of each model in the test set.

Evaluation Indicators	KNN	Decision Tree	Random Forest
MAE	0.27	0.10	0.10
RMSE	0.34	0.12	0.11
R^2^	0.18	0.79	0.90

## Data Availability

The original query data presented in this study can be queried by contacting the author.
